# Statin Intensity or Achieved LDL? Practice-based Evidence for the Evaluation of New Cholesterol Treatment Guidelines

**DOI:** 10.1371/journal.pone.0154952

**Published:** 2016-05-26

**Authors:** Elsie Gyang Ross, Nigam Shah, Nicholas Leeper

**Affiliations:** 1 Division of Vascular Surgery, Stanford University Hospital and Clinics, Stanford, CA, United States of America; 2 Stanford Center for Biomedical Informatics Research, Stanford, CA, United States of America; University of Manitoba, CANADA

## Abstract

**Background:**

The recently updated American College of Cardiology/American Heart Association cholesterol treatment guidelines outline a paradigm shift in the approach to cardiovascular risk reduction. One major change included a recommendation that practitioners prescribe fixed dose statin regimens rather than focus on specific LDL targets. The goal of this study was to determine whether achieved LDL or statin intensity was more strongly associated with major adverse cardiac events (MACE) using practice-based data from electronic health records (EHR).

**Methods:**

We analyzed the EHR data of more than 40,000 adult patients on statin therapy between 1995 and 2013. Demographic and clinical variables were extracted from coded data and unstructured clinical text. To account for treatment selection bias we performed propensity score stratification as well as 1:1 propensity score matched analyses. Conditional Cox proportional hazards modeling was used to identify variables associated with MACE.

**Results:**

We identified 7,373 adults with complete data whose cholesterol appeared to be actively managed. In a stratified propensity score analysis of the entire cohort over 3.3 years of follow-up, achieved LDL was a significant predictor of MACE outcome (Hazard Ratio 1.1; 95% confidence interval, 1.05–1.2; P < 0.0004), while statin intensity was not. In a 1:1 propensity score matched analysis performed to more aggressively control for covariate balance between treatment groups, achieved LDL remained significantly associated with MACE (HR 1.3; 95% CI, 1.03–1.7; P = 0.03) while treatment intensity again was not a significant predictor.

**Conclusions:**

Using EHR data we found that on-treatment achieved LDL level was a significant predictor of MACE. Statin intensity alone was not associated with outcomes. These findings imply that despite recent guidelines, achieved LDL levels are clinically important and LDL titration strategies warrant further investigation in clinical trials.

## Introduction

The American College of Cardiology/American Heart Association (ACC/AHA) recently released updated cholesterol treatment guidelines [1]. These guidelines represent a major departure from established lipid management paradigms [2, 3], and introduced a number of changes affecting both primary and secondary cardiovascular prevention [4, 5].

One major shift in the new guidelines is the recommendation to eschew the current clinical practice of titrating anti-lipid therapy towards specific low-density lipoprotein (LDL) goals. Rather, the new guidelines propose using fixed dose statin therapy based primarily on patient age, clinical characteristics (like diabetes), and risk scores. The guideline authors emphasize that this is a more evidence-based approach given that the randomized clinical trials (RCT) on which the guidelines are based did not explicitly test a hypothesis of statin titration. Concern exists, however, that subjects with known cardiovascular disease who previously had their statins aggressively uptitrated or were prescribed adjunct therapy (like ezetimibe) until a goal LDL of < = 70 mg/dL was achieved [2, 6] may now be treated more conservatively and exposed to higher lipid levels. Furthermore, as other authors have pointed out, with the approval of new lipid lowering therapies, lack of emphasis on achieved LDL may make it difficult for clinicians to make optimal treatment decisions [7].

In light of the debate surrounding these guideline recommendations we set out to use data mining techniques to analyze structured and unstructured data from the electronic health records (EHR) of 19 million clinical encounters to determine whether achieved LDL or statin intensity was a better predictor of adverse cardiovascular events.

## Materials and Methods

The Stanford University Institutional Review Board (IRB) approved this research (Approval ID: 24883). All data used were de-identified and the requirement for informed consent was waived by the IRB.

### Data Source

Data were derived from all patients treated as outpatients and inpatients at Stanford Hospital and Clinics from 1995 to 2013. We utilized the Stanford Translational Research Integrated Database Environment (STRIDE), a clinical data warehouse, that at the time of our analysis included 1.8 million pediatric and adult patients, 19 million clinical encounters, 35 million International Classification of Diseases, ninth revision (ICD-9) codes, and 11 million unstructured clinical notes [8]. Descriptions of how unstructured clinical notes have been processed for use in our text-mining analysis have previously been described [9, 10]. Briefly, the text is processed by mapping terms in the text to medical concepts using different medical dictionaries [11, 12]. Terms with ambiguous meanings are removed, and terms for which there are multiple ways of describing the same concept (e.g. heart attack, myocardial infarction, acute MI, etc.) are collapsed into one concept category. The terms are then contextually analyzed and term mentions corresponding to a patient’s family history or terms that are negated are flagged as such [13, 14]. The resulting data is indexed in such a way that for each patient, and for each note, the terms that appear in the note and their context (applying to the patient, applying to family, or negated) are tabulated and can be extracted for further analysis.

### Study Group

**Fig 1:**
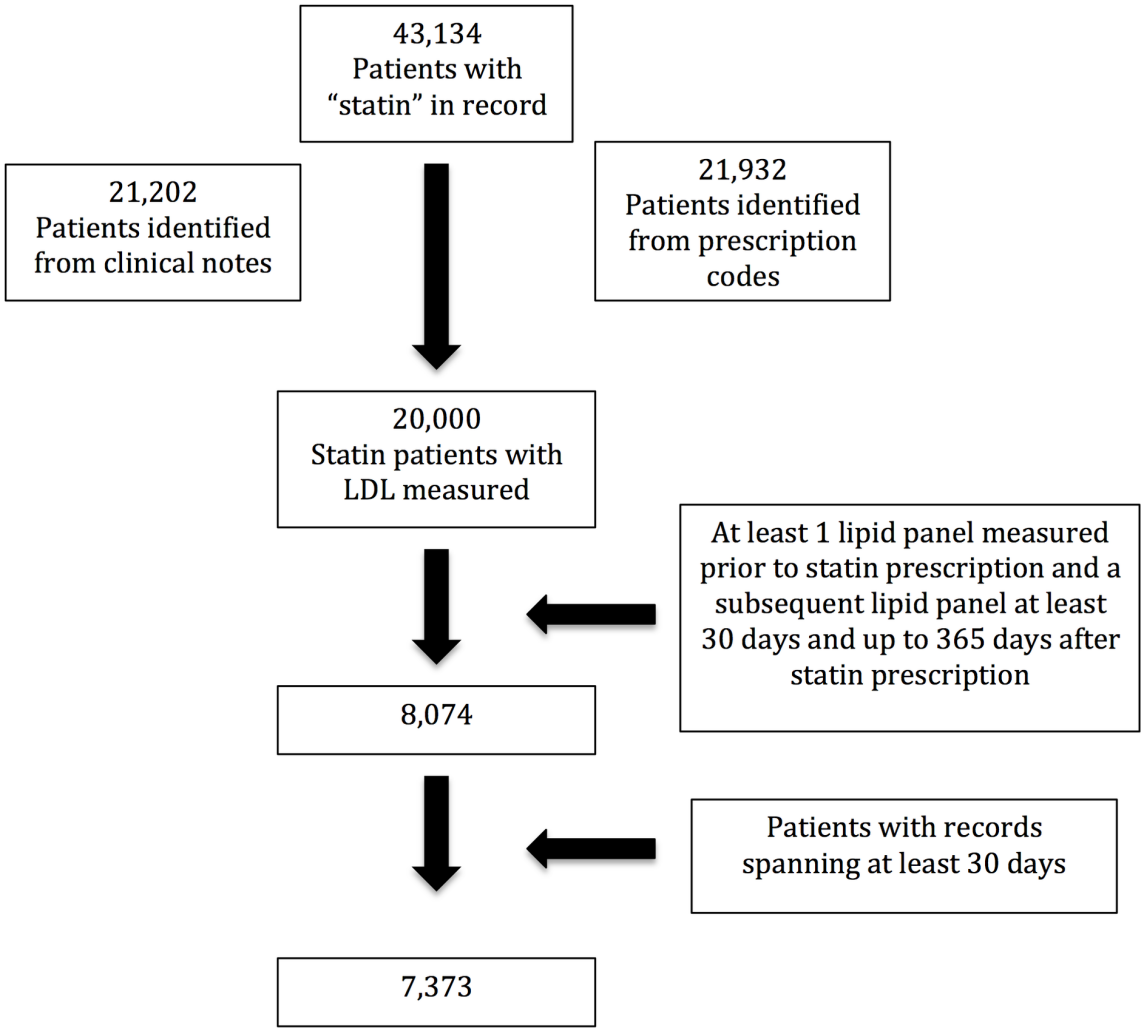
Inclusion/Exclusion criteria. LDL, low-density lipoprotein.

Using coded data and our text-mining pipeline we identified all patients prescribed high-, moderate- and low-intensity statin therapy [1] over the age of 21 years (Table 1). Given the nature of our institution as a tertiary care center, we anticipated that many patients would be noted to be on a statin drug, whether or not they were receiving active management of their cholesterol levels. Since guideline recommendations would mostly affect patients whose cholesterol levels are actively managed, we excluded those who did not have a lipid panel measured before statin therapy appeared in their medical record as well as those who did not have a lipid panel measured 30 days to 1 year after statin therapy appeared in their record (Fig 1). This time frame was chosen based on examination of the pharmacodynamics of a wide range of statins [15]. We also excluded patients whose medical follow-up records ended before 30 days.

**Table 1:** Statin therapy dosage and intensity (from ACC/AHA Guidelines)^a^.

High-Intensity Statin	Moderate-Intensity Statin	Low-Intensity Statin
Atorvastatin 40–80 mg	Atorvastatin 10–20 mg	Simvastatin 10 mg
Rosuvastatin 20–40 mg	Rosuvastatin 5–10 mg	Pravastatin 10–20 mg
	Simvastatin 20–40 mg	Lovastatin 20 mg
	Pravastatin 40–80 mg	Fluvastatin 20–40 mg
	Lovastatin 40 mg	Pitavastatin 1 mg
	Fluvastatin XL 80 mg	
	Fluvastatin 40 mg bid	
	Pitavastatin 2–4 mg	

^a^From: Stone NJ, Robinson JG, Lichtenstein AH et al. 2013 ACC/AHA Guideline on the Treatment of Blood Cholesterol to Reduce Atherosclerotic Cardiovascular Risk in Adults: A Report of the American College of Cardiology/American Heart Association Task Force on Practice Guidelines. Journal of the American College of Cardiology 2014;63:2889–934.

Of the patients selected, we collected demographic information as well as their comorbidities and co-prescriptions (S1 Table). We used major cardiac events such as myocardial infarction, cardiac arrest, defibrillation events, stroke, and sudden death as our composite outcome measure. These disease states were chosen for our MACE definition as they could be reliably extracted from the EHR with high precision and recall. ICD-9 codes and text-based concept codes used to define MACE outcomes are provided in S2 and S3 Tables.

### Stratified Propensity Score Analysis

In order to account for statin treatment selection bias, while also using the full complement of patient data, we performed a stratified propensity score analysis. Using a multinomial logistic regression model we derived propensity scores for patients treated with high-, moderate- and low-intensity statin therapy, adjusting for patient age, gender, race, ethnicity, comorbidities, co-prescriptions and pre-statin cholesterol levels. Patients were then divided into multiple strata based on their propensity scores. We tested covariate balance using between 3–10 strata and chose the number of strata providing the best intra-strata balance as recommended [16]. Balance was assessed by comparing proportions and mean difference between groups using a P-value significance level of 0.05.

### 1:1 Propensity Score Matching

For more aggressive control of covariate balance between treatment groups, while minimizing confounding bias for treatment selection, we also performed 1:1 propensity score matched analysis [17, 18]. An initial analysis demonstrated that patients treated with high-intensity statins had significant differences in underlying characteristics as compared with those on low- and moderate-intensity statins (Table 2), thus in our 1:1 propensity score analysis patients treated with high-intensity statins were the treatment group and patients receiving low- and moderate-intensity statins were collapsed into the control group. Using the Matching package in R [19] we derived propensity scores using a logistic regression model that included age, gender, race, ethnicity, comorbidities, co-prescriptions and pre-statin cholesterol levels. We also matched patients based on total follow-up time to take into account that patients followed for longer intervals of time are more likely to eventually have a MACE outcome. Once propensity scores were derived, we performed 1:1 propensity score matching without replacement.

**Table 2:** Demographic and Clinical Data for 7,373 patients.

	Low	Moderate	High	P-value
**N**	1,355	4,990	1,028	
**Age (mean years)**	62.8 ± 14	63.8 ± 14	65 ± 12	0.004
**Sex (%)**				
M	51	56	65	7.5e-11
F	49	44	35	7.5e-11
**Race (%)**				
Caucasian	54	54	61	0.0006
African-American	5	5	5	0.7
Other^a^	41	41	34	0.003
**Ethnicity (%)**				
Hispanic	7	7	7	0.7
**Comorbidities (%)**				
Coronary artery disease	52	56	72	< 2.2e-16
Congestive heart failure	37	40	57	< 2.2e-16
Chronic kidney disease	24	22	25	0.04
Type 2 Diabetes	64	63	66	0.3
Hypertension	86	84	90	2.1e-06
Peripheral artery disease	30	30	43	7.8e-15
Previous MACE ^b^	51	52	67	<2.2e-16
**Co-prescriptions (%)**				
ACE-Inhibitors/ARBs	56	59	71	5.7e-13
Aspirin	74	75	88	< 2.2e-16
Beta-blockers	52	55	70	< 2.2e-16
Statin Adjuncts	20	18	30	3.9e-16

^a^Other refers to East Asians, South Asians, Native Americans and those who specifically report “other” in their demographic profiles.

^b^Previous major adverse cardiac event including myocardial infarction, stroke, cardiac arrest, defibrillation events.

MACE, major adverse cardiac event; ACE, angiotensin-converting enzyme; ARBs, angiotensin II receptor blockers.

### Statistical Analysis

For demographic variables analysis of variance (ANOVA) or the Kruskal-Wallis tests were used for continuous variables depending on whether the sample characteristic being tested was normally distributed. Chi Squared tests were used for categorical variables. To account for censoring in the data we used a multivariate Cox proportional hazards model stratified by matched pairs and propensity score-based strata to evaluate characteristics associated with risk of MACE outcome [20].

### Enrichment Analysis

In genomics research enrichment analysis is a technique used to gain insight into the biological function of a subset of genes. Once a group of genes has been identified as being significantly up-regulated in a population of interest, these genes can then be annotated using a dictionary of controlled terms (called the Gene Ontology [21]) linking gene expression to biological processes, thus providing functional insights [22]. As an analogous descriptive analysis, we set out to identify concepts that were differentially mentioned, and “enriched” in the clinical records of patients with MACE outcomes. To do this terms from the patients' medical records were mapped to the Unified Medical Language Systems [23], which allowed us to collapse multiple terms into concepts and subsequently into broad concept categories [24]. We then used the Fisher's exact test to identify whether the frequency of these concepts were significantly different between patients with and without a MACE outcome. The results are visualized as a word cloud using Wordle (www.wordle.net) to create a descriptive view of the patient groups. In general, such analysis can provide key insights into different patient groups and the terms, diseases and events that are associated with their disease state.

## Results

We found a total of 43,134 adult patients who had been taking a statin (Table 1) at some point during their care at our institution. Of these patients 7,373 had their statins actively managed (defined as having at least 1 lipid panel measurement before statin initiation and another measurement 4 weeks to a year after therapy began) (Fig 1).

Table 2 shows the breakdown of demographic and clinical data by statin intensity. Mean age across all groups was 64 years. Fifty-six percent of all patients were male and 55% were Caucasian. Overall, patients had a high burden of disease– 85% had hypertension (HTN), 64% had Type 2 diabetes, 58% had coronary artery disease (CAD), 42% had congestive heart failure (CHF), and 32% had peripheral artery disease (PAD). Mean patient follow-up was 3.3 ± 2.4 years. A total of 1,115 patients experienced a MACE outcome. Patients treated with high-intensity statins differed significantly from those on low- and moderate-intensity statins. Patients on high-intensity statins were significantly older, more likely to be male, Caucasian, and in general had a higher burden of disease and were on more medications.

### Stratified Propensity Score Analysis

In our stratified propensity score model, patients were overall well balanced within 5 strata with only minor areas of imbalance (S4 Table). Achieved cholesterol profiles of patients by stratum are presented in the S5 Table.

Using a stratified Cox proportional hazards model adjusting for demographic and clinical factors (age, sex, race, comorbidities, and co-prescriptions), we found that higher achieved LDL was associated with higher risk of MACE outcome (Hazard Ratio (HR) 1.1; 95% confidence interval (CI), 1.05–1.2; P = 0.0004) (S6 Table, S1 Fig), while statin intensity alone was not associated with MACE outcome (P = NS). Since LDL was scaled in our models the HR can be interpreted as the following—with a mean achieved LDL of 95 mg/dL and standard deviation (SD) of 31 mg/dL, for every SD increase in achieved LDL the annual risk of having a MACE event increased by 10%. Furthermore, a history of congestive heart failure was associated with higher risk of MACE outcome (HR 1.2; 95% CI, 1.0–1.4; P = 0.04) while being on aspirin therapy had a significant, albeit small, protective effect (HR 0.8; 95% CI, 0.7–0.99; P = 0.047). Race and other demographic and clinical factors were not significantly associated with MACE outcomes.

### 1:1 Propensity Score Matching Analysis

**Fig 2:**
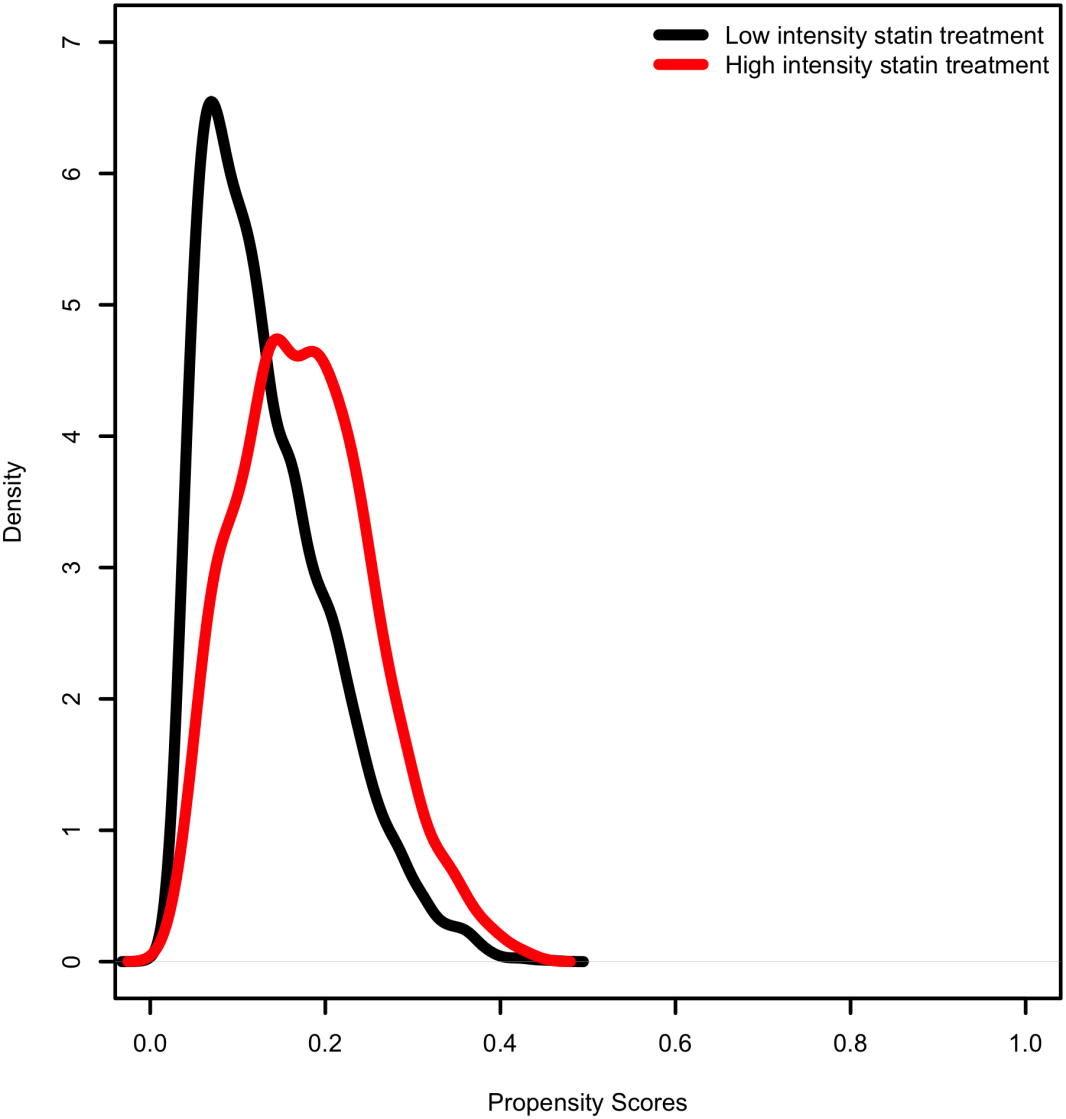
Propensity score distribution *before* matching.

**Fig 3:**
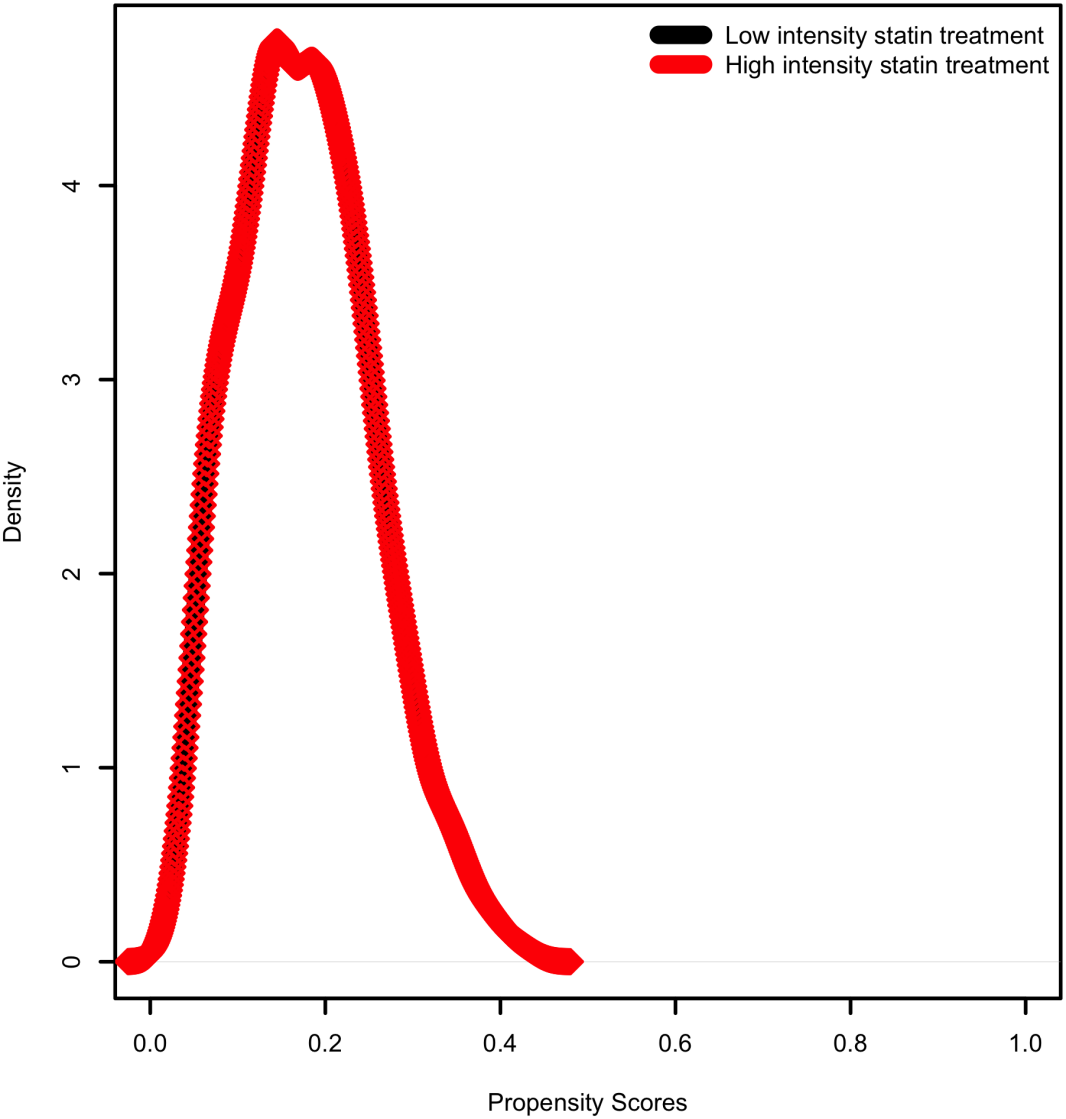
Propensity score distribution *after* matching.

Propensity matched analysis produced 1,028 matched pairs of patients on high-intensity statins matched to those on low- or moderate intensity statins for a total of 2,056 patients. A total of 281 patients had a MACE outcome in this matched cohort. After matching there were no significant differences between the two groups on demographic or clinical factors (Table 3). The distributions of propensity scores before and after matching are illustrated in Figs 2 and 3. Mean achieved LDL in the matched cohort was 88 ± 30 mg/dL. Mean HDL and triglycerides were 49 ± 15 mg/dL and 125 ± 83 mg/dL, respectively.

**Table 3:** Demographic and clinical characteristics of matched patient cohort (N = 2,056).

	Low-/Moderate-intensity treatment	High-intensity treatment	P-value
**N**	1,028	1,028	
**Age (mean years)**	65	65	0.08
**Sex (%)**			
M	66	65	0.4
F	34	35	0.4
**Race (%)**			
Caucasian	61	61	0.8
African-American	4	5	0.5
Other^a^	34	34	0.6
**Ethnicity (%)**			
Hispanic	6.5	6.5	1
**Comorbidities (%)**			
Coronary artery disease	67	67	0.8
Congestive heart failure	55	57	0.9
Chronic kidney disease	24	25	0.7
Type 2 Diabetes	66	66	0.7
Hypertension	90	90	0.9
Peripheral artery disease	44	43	0.4
Previous MACE	67	67	0.8
**Co-prescriptions (%)**			
ACE-Inhibitors/ARBs	70	70	0.6
Aspirin	89	88	0.2
Beta-blockers	68	70	0.2
Statin Adjuncts	22	24	0.2

^a^Other refers to East Asians, South Asians, Native Americans and those who specifically report “other” in their demographic profiles.

MACE, major adverse cardiac event; ACE, angiotensin-converting enzyme; ARBs, angiotensin II receptor blockers; LDL, low-density lipoprotein; HDL, high-density lipoprotein.

**Fig 4:**
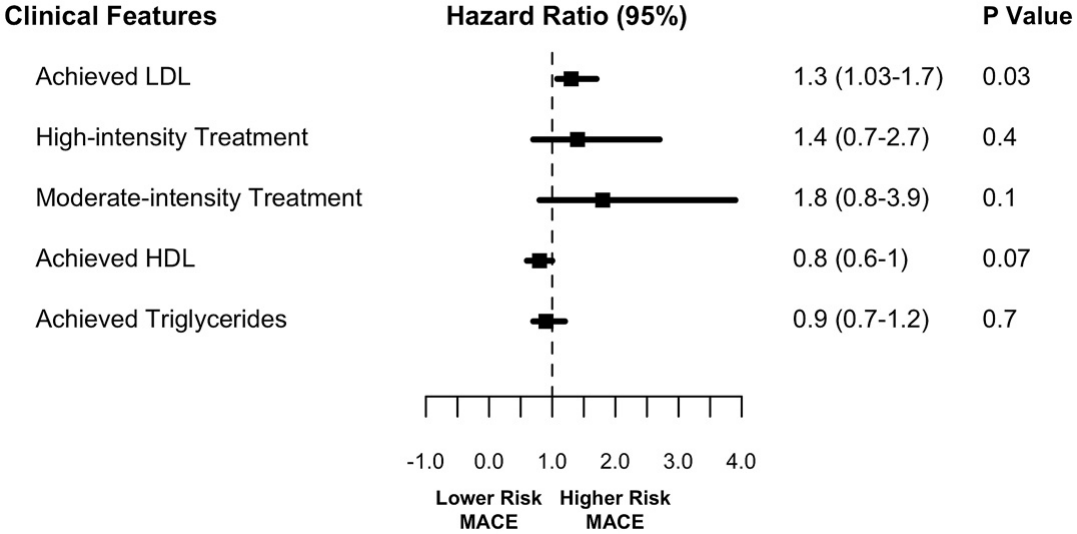
Hazard ratios for cholesterol levels and intensity of statin therapy in matched cohort (N = 2,056). LDL, low-density lipoprotein; HDL, high-density lipoprotein.

**Fig 5:**
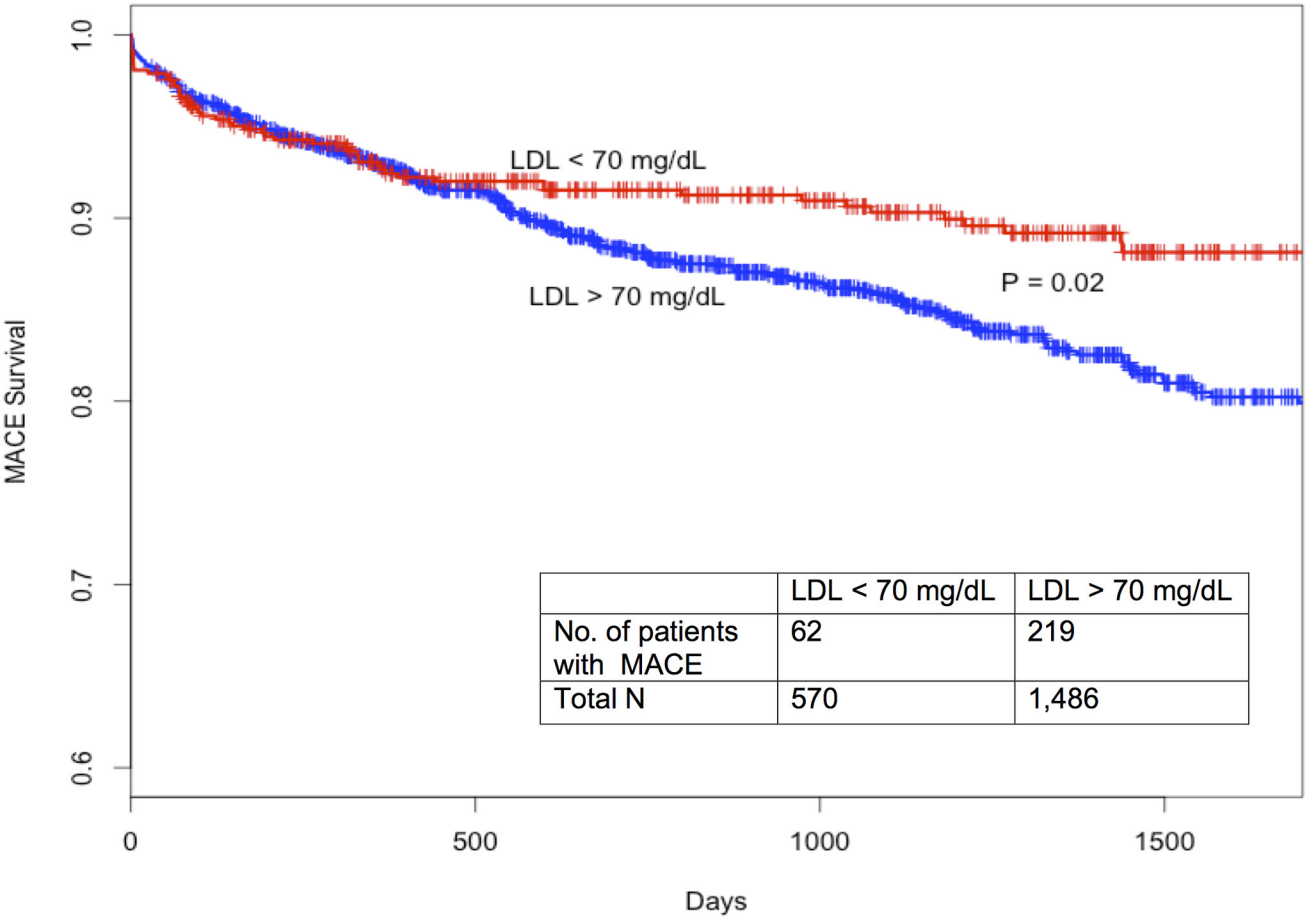
Kaplan-Meier freedom from major adverse cardiac events plot comparing patients with LDL above and below 70 mg/dL in matched cohort (N = 2,056). LDL, low-density lipoprotein; MACE, major adverse cardiac event.

Using a Cox proportional hazards model stratified by matched pairs (Table 4) and adjusting for demographic and clinical factors (age, sex, race, comorbidities, and co-prescriptions), we again found that higher achieved LDL was associated with a higher risk of MACE (HR 1.3; 95% CI, 1.03–1.7; P = 0.03) while statin intensity was not (P = NS) (Fig 4). Other demographic and clinical factors were not significantly associated with MACE outcomes. A Kaplan Meier plot showing survival differences based on LDL level greater than or less than 70 mg/dL is illustrated in Fig 5.

**Table 4:** Stratified Cox proportional hazards model^a^ of MACE outcomes in matched cohort (N = 2,056).

	Hazard Ratio [95% CI]	P-value
**Achieved LDL**^b^	**1.3 [1.03, 1.7]**	**0.03**
High-Intensity Treatment	1.4 [0.7, 2.7]	0.4
Moderate-Intensity Treatment	1.8 [0.8, 3.9]	0.1
Low-Intensity Treatment	Reference	
HDL^c^	0.8 [0.6, 1.0]	0.07
Triglycerides^d^	0.9 [0.7, 1.2]	0.7

^a^Adjusted for age, gender, race, ethnicity, history of coronary artery disease, congestive heart failure, chronic kidney disease, hypertension, peripheral artery disease, Type 2 diabetes, and co-prescriptions including angiotensin-converting enzyme inhibitors/angiotensin II receptor blockers, aspirin, beta-blockers, and statin adjuncts.

^b^Scaled variable. Center LDL = 88 ± 30. Therefore for each 30 mg/dL increase in achieved LDL the hazard rate for MACE increases by 30%.

^**c**^Scaled variable. Center HDL = 49 ± 15.

^**d**^Scaled variable. Center Triglyceride = 125 ± 83 mg/dL.

MACE, major adverse cardiac event; LDL, low-density lipoprotein; HDL, high-density lipoprotein.

### Enrichment Analysis

**Fig 6:**
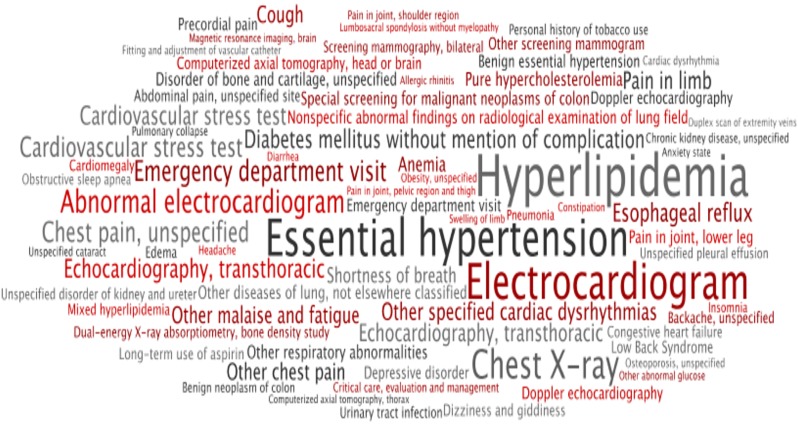
Word cloud of top 75 words enriched in patients having a major adverse cardiac event during follow-up compared to those who did not (N = 7,373).

Those who had a MACE outcome during follow-up in our cohort of 7,373 patients tended to have enrichment (i.e. higher frequency) of concepts related to high acuity cardiovascular disease including emergency room visits, chest pain, shortness of breath, catheterization and echocardiography procedures as well as a history of pulmonary related conditions such as “tobacco use disorder” and obstructive sleep apnea (Fig 6). They also had enrichment of comorbidities such as diabetes, hypertension, kidney disease, obesity and anemia.

## Discussion

Given the controversy associated with the new ACC/AHA lipid management guidelines, we pursued an approach of using EHR data to gain insights into factors associated with cardiovascular outcomes. We found that simply being on a higher potency statin was not associated with improved outcomes, whereas achieved LDL was a significant predictor of MACE. These findings were observed using two different types of propensity score analyses employed to reduce treatment selection bias. Taken together, these data suggest that explicit LDL targets may still have a role in clinical practice.

Since the seminal publication of epidemiologic data from the Framingham Heart Study, total serum cholesterol level has been considered an important marker of cardiovascular health and a major target for preventing cardiovascular events [25]. The Seven Countries study, which spanned multiple countries and cultures, corroborated results from the Framingham study and demonstrated a linear relationship between serum cholesterol and cardiovascular events [26]. Further, clinical trials of statin therapy spanning multiple decades and including thousands of patients show that reducing cholesterol levels can significantly improve patient outcomes [27–30]. Such strong epidemiological evidence and trial data have formed the basis for lipid management recommendations put forth by some of the largest cardiovascular health organizations including the Adult Treatment Panel and the European Society of Cardiology. In addition to focusing on lifestyle modifications, recommendations from these organizations have historically focused on targets for LDL levels depending on patient risk factors.

What has been up for more recent debate is whether specific LDL goals are warranted given best evidence. In aggregate, trial data seem to suggest that targeting lower LDL levels for high-risk patients may provide the best long-term outcomes. The Cholesterol Treatment Trialists’ Collaboration (CTTC) published work in both 2005 and 2010 highlighting the incremental benefit of decreasing LDL [31, 32]. Their first publication was a meta-analysis of 14 large RCTs that included over 90,000 patients who were followed for an average of 5 years. The goal of their analysis was to estimate how clinical outcomes varied per 1.0 mmol/L (1.6 mg/dL) reduction in LDL. Findings were that a modest 1.0 mmol/L reduction in LDL was associated with a 20% reduction in the annual risk of a major cardiovascular event. For patients with a history of coronary heart disease this amounted to 48 fewer patients per 1000 having a major cardiovascular event over that 5-year time period. In 2010, the CTTC published another meta-analysis examining the effects of lipid lowering beyond “standard” thresholds. In a total of 26 statin therapy trials that followed almost 170,000 patients for an average of 5 years, each 1 mmol/L reduction in LDL produced an annual 10% reduction in risk of all-cause mortality. Despite more intensive lipid lowering, the authors did not find a significant increase in death from cancer or other non-vascular causes.

In addition to the clinical trial and epidemiology data provided above, newer genetic data also provide a rationale for the aggressive treatment of LDL. For example, studies employing Mendelian Randomization have provided strong evidence that LDL is not only *associated with* cardiovascular disease, but also *causal for* cardiovascular disease [33]. This distinction is important, as it has helped explain why drugs that raise HDL (which is no longer considered causal for myocardial infarction [33]) have failed in the past, and why LDL-lowering drugs have proven so effective.

Additional support for the ‘lower is better’ approach is provided by results of the IMPROVE-IT study [34], where ezetimibe added to simvastatin therapy was associated with improved clinical outcomes, even after titration of the statin dose based on the achieved LDL. While it is possible that the ezetimibe provided this benefit through some unknown mechanism, it is likely that the lower achieved LDL level in this group (54 mg/dL vs 70 mg/dL) was the driving force associated with the improved outcomes. In this regard, our prior studies have suggested that aggressive lipid-lowering therapy continues to be associated with improved survival even amongst those with extremely low cholesterol levels, including individuals with LDLs less than 40 mg/dL [35].

At the time of writing, the ACC/AHA’s position was that recommendations for specific LDL targets could not be made given that no RCTs had expressly tested the hypothesis that titrating to a specific LDL goal was more beneficial than simply placing patients on a tiered intensity statin. However, basing recommendations solely on availability of RCT data can be problematic. As Lopez-Jimenez and colleagues point out in their assessment of the 2013 ACC/AHA Guidelines, RCTs often exclude large swaths of patients for whom a clinical question is expressly relevant and can be limited by short follow-up time horizons [4]. An advantage to our informatics approach of using EHR data is that we are able to search for effects in all patients placed on statins, regardless of their clinical phenotype.

Overall, our findings demonstrate that electronic health records can serve as a valuable source of data for addressing questions regarding clinical equipoise. Even so, this study has some limitations. First, this is an analysis of retrospective, observational data and thus despite our attempts to control for treatment selection bias through propensity score analyses, there may be confounding variables for which we did not or could not control. For instance, we cannot tease out patient compliance with statin therapy, which may have an effect on our observations. Other confounding factors may include comorbidities and medications that we did not explicitly extract from our data that may influence outcomes. Other variables that could also influence outcomes that we did not have access to given the nature of EHR data include patient diet, nutritional status, and physical activity.

Another limitation is that we could not quantify the Pooled Cohort Equations risk scores, a new risk calculator developed by the ACC/AHA Risk Assessment Work Group and used in the new guidelines, due to our current inability to extract smoking status and specific blood pressure measurements from our EHR. Thus, we could not directly test the performance of the new guidelines in comparison to prior Adult Treatment Panel III guidelines in regards to risk stratification and statin intensity recommendations. In this same vein we were unable to extract disease severity in patients with CHF, which may have an effect on the magnitude to which this disease category influences risk of MACE. Furthermore, our definition of our MACE outcome variable was based on terms that could reliably be extracted and did not include other potential outcomes of interest such as unstable angina.

## Conclusion

Mining data from our EHR revealed that achieved LDL is significantly associated with MACE outcome while intensity of statin therapy is not. Although the 2013 ACC/AHA guidelines advise clinicians to abandon the clinical practice of targeting specific LDL thresholds with statin therapy, our findings in conjunction with the findings of multiple meta-analyses and Mendelian Randomization studies highlight the importance of specific LDL levels and their modulatory effect on cardiovascular events. Concern persists that abandoning the focus on specific LDL levels may result in preventable cardiac events in high-risk patients. These data provide additional support for an RCT that compares the outcomes associated with the approach of targeting LDL levels explicitly, compared with an approach based on statin intensity alone.

## Supporting Information

S1 Fig Hazard ratios for cholesterol levels, statin intensity and clinical factors related to MACE outcome in stratified propensity score analysis (N = 7,373). LDL—low-density lipoprotein; HDL—high-density lipoprotein.
(TIF)

S1 Table Definition of demographic, comorbidities, co-prescriptions and outcome variables collected for each patient. ACE Inhibitors—angiotensin-converting enzyme inhibitors; ARBs—angiotensin II receptor blockers; MACE, major adverse cardiac event.
(DOCX)

S2 Table International Classification of Diseases, Ninth Revision codes used to identify MACE outcome. MACE, major adverse cardiac event; AMI, acute myocardial infarction; NEC, not elsewhere classified; NOS, not otherwise specified.
(DOCX)

S3 Table Concept terms and codes used to identify MACE outcome. MACE, major adverse cardiac event.
(DOCX)

S4 Table Demographic and clinical characteristics of patients treated with high intensity statins versus low or moderate intensity statins by strata (N = 7,373). ^a^Treated refers to patients on high-intensity statin therapy. Control refers to patients on moderate- or low-intensity statin therapy. CAD, coronary artery disease; CHF, congestive heart failure; CKD, chronic kidney disease; PAD, peripheral artery disease; MACE, major adverse cardiac event; ACE-I, angiotensin-converting enzyme inhibitor; ARBs, angiotensin II receptor blockers.
(DOCX)

S5 Table Cholesterol profiles of patients treated with high intensity statins versus low or moderate intensity statins by strata (N = 7,373). ^a^Treated refers to patients on high-intensity statin therapy. Control refers to patients on moderate- or low-intensity statin therapy. LDL, low-density lipoprotein; HDL, high-density lipoprotein.
(DOCX)

S6 Table Stratified Cox proportional hazards model^a^ of MACE Outcomes based on patient characteristics. ^a^Adjusted for age, gender, race, ethnicity, history of coronary artery disease, congestive heart failure, chronic kidney disease, hypertension, peripheral artery disease, Type 2 diabetes, and co-prescriptions including angiotensin-converting enzyme inhibitors/angiotensin II receptor blockers, aspirin, beta-blockers, and statin adjuncts. ^b^Scaled variable. Center LDL = 95 ± 31. Therefore for each 31 mg/dL increase in achieved LDL the hazard rate for MACE increases by 10%. ^c^Scaled variable. Center HDL = 51 ± 16. ^d^Scaled variable. Center Triglyceride = 127 ± 79 mg/dL. MACE, major adverse cardiac event; LDL, low-density lipoprotein; HDL, high-density lipoprotein.
(DOCX)
