# Supplementary figures and images for: Statin Intensity or Achieved LDL? Practice-based Evidence for the Evaluation of New Cholesterol Treatment Guidelines
Source: PLoS One. 2016 May 26;11(5):e0154952. doi: 10.1371/journal.pone.0154952 (PMC4881915; doi:10.1371/journal.pone.0154952)

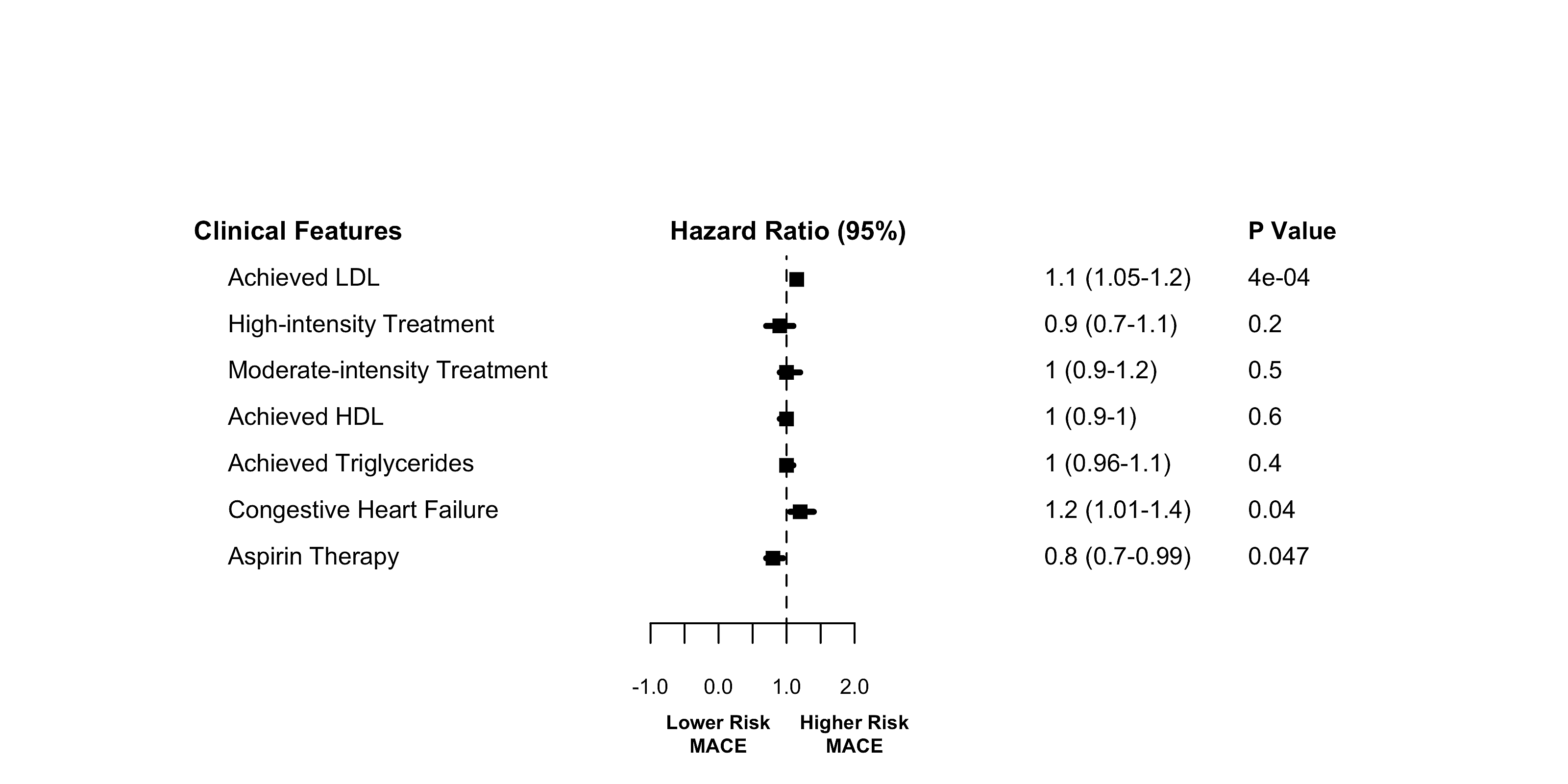

Supplement: S1 Fig — LDL—low-density lipoprotein; HDL—high-density lipoprotein. (TIF) [file pone.0154952.s001.tif]
