# Supplementary material for: Statin Intensity or Achieved LDL? Practice-based Evidence for the Evaluation of New Cholesterol Treatment Guidelines
Source: PLoS One. 2016 May 26;11(5):e0154952. doi: 10.1371/journal.pone.0154952 (PMC4881915; doi:10.1371/journal.pone.0154952)
Supplement: S1 Table — ACE Inhibitors—angiotensin-converting enzyme inhibitors; ARBs—angiotensin II receptor blockers; MACE, major adverse cardiac event. (DOCX) [file pone.0154952.s002.docx]

S1 Table. Definition of demographic, comorbidities, co-prescriptions and outcome variables collected for each patient.

| Demographics | Comorbidities | Co-prescriptions | Outcomes |
| --- | --- | --- | --- |
| Age | Coronary artery disease | ACE Inhibitors/ARBs | **MACE** |
| Gender | Congestive heart failure | Aspirin | Cardiac Arrest |
| Race | Chronic kidney disease | Beta-blockers | Defibrillation events |
| Ethnicity | Hypertension | **Statin Adjuncts** | Myocardial infarction |
|  | Peripheral artery disease  Type 2 Diabetes | Ezetimibe  Niacin | Stroke  Sudden cardiac death |

ACE Inhibitors – angiotensin-converting enzyme inhibitors; ARBs – angiotensin II receptor blockers; MACE, major adverse cardiac event.
