# Supplementary material for: Statin Intensity or Achieved LDL? Practice-based Evidence for the Evaluation of New Cholesterol Treatment Guidelines
Source: PLoS One. 2016 May 26;11(5):e0154952. doi: 10.1371/journal.pone.0154952 (PMC4881915; doi:10.1371/journal.pone.0154952)
Supplement: S2 Table — MACE, major adverse cardiac event; AMI, acute myocardial infarction; NEC, not elsewhere classified; NOS, not otherwise specified. (DOCX) [file pone.0154952.s003.docx]

**S2 Table.** **International Classification of Diseases, Ninth Revision codes used to identify MACE outcome**

| ICD 9 Codes | Code string |
| --- | --- |
| 410 | Acute myocardial infarction |
| 410.0 | Acute myocardial infarction, of anterolateral wall |
| 410.00 | AMI anterolateral, unspecified |
| 410.01 | AMI anterolateral, initial |
| 410.02 | AMI anterolateral, subsequent |
| 410.1 | Acute myocardial infarction, of other anterior wall |
| 410.10 | AMI anterior wall, unspecified |
| 410.11 | AMI anterior wall, initial |
| 410.12 | AMI anterior wall, subsequent |
| 410.2 | Acute myocardial infarction, of inferolateral wall |
| 410.20 | AMI inferolateral, unspecified |
| 410.21 | AMI inferolateral, init |
| 410.22 | AMI inferolateral, subsequent |
| 410.3 | Acute myocardial infarction, of inferoposterior wall |
| 410.30 | AMI inferoposterior, unspecified |
| 410.31 | AMI inferoposterior, initial |
| 410.32 | AMI inferoposterior, subsequent |
| 410.4 | Acute myocardial infarction, of other inferior wall |
| 410.40 | AMI inferior wall, unspecified |
| 410.41 | AMI inferior wall, initial |
| 410.42 | AMI inferior wall, subsequent |
| 410.5 | Acute myocardial infarction, of other lateral wall |
| 410.50 | AMI lateral NEC, unspecified |
| 410.51 | AMI lateral NEC, initial |
| 410.52 | AMI lateral NEC, subsequent |
| 410.6 | Acute myocardial infarction, true posterior wall infarction |
| 410.60 | True posterior infarct, unspecified |
| 410.61 | True posterior infarct, initial |
| 410.62 | True posterior infarct, subsequent |
| 410.7 | Acute myocardial infarction, subendocardial infarction |
| 410.70 | Subendocardial infarct, unspecified |
| 410.71 | Subendocardial infarct, initial |
| 410.72 | Subendocardial infarct, subsequent |
| 410.8 | Acute myocardial infarction, of other specified sites |
| 410.80 | AMI NEC, unspecified |
| 410.81 | AMI NEC, initial |
| 410.82 | AMI NEC, subsequent |
| 410.9 | Acute myocardial infarction, unspecified site |
| 410.90 | AMI NOS, unspecified |
| 410.91 | AMI NOS, initial |
| 410.92 | AMI NOS, subsequent |
| 427.4 | Ventricular fibrillation and flutter |
| 427.41 | Ventricular fibrillation |
| 427.42 | Ventricular flutter |
| 427.5 | Cardiac arrest |
| 785.51 | Cardiogenic shock |
| 99.6 | Conversion of cardiac rhythm |
| 99.60 | Cardiopulmonary resuscitation NOS |
| 99.62 | Heart counter shock NEC |
| 99.63 | Closed chest cardiac massage |
| 99.69 | Cardiac rhythm conversion NEC |

MACE, major adverse cardiac event; AMI, acute myocardial infarction; NEC, not elsewhere classified; NOS, not otherwise specified.
