# Supplementary material for: Statin Intensity or Achieved LDL? Practice-based Evidence for the Evaluation of New Cholesterol Treatment Guidelines
Source: PLoS One. 2016 May 26;11(5):e0154952. doi: 10.1371/journal.pone.0154952 (PMC4881915; doi:10.1371/journal.pone.0154952)
Supplement: S3 Table — MACE, major adverse cardiac event. (DOCX) [file pone.0154952.s004.docx]

**S3 Table.** **Concept terms and codes used to identify MACE outcome**

| Concept String | Concept Unique Identifier |
| --- | --- |
| cerebral infarction | C0007785 |
| multi-infarct dementia | C0011263 |
| electric countershock | C0013778 |
| cardiac arrest | C0018790 |
| myocardial infarction | C0027051 |
| shock, cardiogenic | C0036980 |
| cerebrovascular accident | C0038454 |
| lateral medullary syndrome | C0043019 |
| sudden cardiac death | C0085298 |
| acute myocardial infarction | C0155626 |
| acute myocardial infarction of anterolateral wall | C0155627 |
| atrial cardioversion | C0199550 |
| multi-infarct dementia, uncomplicated | C0236650 |
| acute anteroseptal myocardial infarction | C0264699 |
| acute inferior myocardial infarction | C0264700 |
| acute myocardial infarction of inferolateral wall | C0340308 |
| silent myocardial infarction | C0340324 |
| subsequent myocardial infarction | C0348593 |
| internal cardioversion | C0419060 |
| circulatory arrest | C0444720 |
| non-q wave myocardial infarction | C0542269 |
| direct current cardioversion | C0542380 |
| multiple lacunar infarcts | C0585229 |
| right sided cerebral hemisphere cerebrovascular accident | C0586324 |
| left sided cerebral hemisphere cerebrovascular accident | C0586325 |
| postoperative myocardial infarction | C0589368 |
| cardiopulmonary arrest | C0600228 |
| cardioembolic stroke | C1531624 |
| acute anterior wall myocardial infarction | C2349195 |

MACE, major adverse cardiac event.
