# Supplementary material for: Statin Intensity or Achieved LDL? Practice-based Evidence for the Evaluation of New Cholesterol Treatment Guidelines
Source: PLoS One. 2016 May 26;11(5):e0154952. doi: 10.1371/journal.pone.0154952 (PMC4881915; doi:10.1371/journal.pone.0154952)
Supplement: S4 Table — aTreated refers to patients on high-intensity statin therapy. Control refers to patients on moderate- or low-intensity statin therapy. CAD, coronary artery disease; CHF, congestive heart failure; CKD, chronic kidney disease; PAD, peripheral artery disease; MACE, major adverse cardiac event; ACE-I, angiotensin-converting enzyme inhibitor; ARBs, angiotensin II receptor blockers. (DOCX) [file pone.0154952.s005.docx]

S4 Table. Demographic and clinical characteristics of patients treated with high-intensity statins versus low- or moderate-intensity statins by strata (N = 7,373)

|  | Strata 1  N= 1,475  Treated^a^/Control | *P-value* | Strata 2  N = 1,474  Treated/Control | *P-value* | Strata 3  N = 1,475  Treated/Control | *P-value* | Strata 4  N = 1,474  Treated/ Control | *P-value* | Strata 5  N = 1,475  Treated/Control | *P-value* |
| --- | --- | --- | --- | --- | --- | --- | --- | --- | --- | --- |
| Age (mean years) | 61 / 59 | 0.7 | 61 / 62 | 0.2 | 64 / 64 | 0.8 | 67 / 66.5 | 0.3 | 66 / 67 | 0.06 |
| Male (%) | 43 / 33 | 0.09 | 46 / 49 | 0.5 | 55 / 54 | 0.8 | 64 / 63 | 0.9 | 81 / 81 | 1 |
| Race (%) |  |  |  |  |  |  |  |  |  |  |
| Caucasian | 42 / 41 | 0.9 | 48 / 51 | 0.5 | 51 / 54 | 0.5 | 58 / 60 | 0.5 | 76 / 70 | **0.02** |
| African-American | 7 / 3 | 0.2 | 9 / 4 | 0.08 | 6 / 5 | 0.6 | 4 /6 | 0.06 | 4 / 5.5 | 0.2 |
| Other | 51 / 56 | 0.6 | 44 / 45 | 0.2 | 43 / 42 | 0.07 | 39 / 34 | 0.5 | 20 / 25 | 0.2 |
| Ethnicity (%) |  |  |  |  |  |  |  |  |  |  |
| Hispanic | 7 / 7 | 0.9 | 13 / 16 | **0.04** | 5 / 8 | 0.07 | 7 / 6 | 0.3 | 4.5 / 7 | 0.1 |
| Comorbidities (%) |  |  |  |  |  |  |  |  |  |  |
| CAD | 18 / 16 | 0.7 | 31 / 37 | 0.1 | 62 / 61 | 0.7 | 82 / 81 | 0.5 | 95 / 94 | 0.3 |
| CHF | 5 / 7 | 0.6 | 22 / 18 | 0.3 | 42 / 37 | 0.2 | 64 / 65 | 0.7 | 82 / 83 | 0.6 |
| CKD | 18 / 14 | 0.3 | 21 / 18 | 0.5 | 22 / 23 | 0.6 | 32 / 32 | 0.9 | 25 /27 | 0.3 |
| Type 2 Diabetes | 49 / 48 | 0.9 | 59 / 62 | 0.5 | 62 / 67 | 0.2 | 70 / 70 | 0.9 | 70 / 72 | 0.3 |
| Hypertension | 71 / 64 | 0.2 | 82 / 83 | 0.8 | 89 / 89 | 0.8 | 92 / 94 | 0.3 | 96 / 95 | 0.5 |
| PAD | 12 / 6 | 0.09 | 18 / 16 | 0.5 | 36 / 31 | 0.2 | 46 / 45 | 0.7 | 59 / 64 | 0.09 |
| Previous MACE | 22 / 15 | 0.1 | 36 / 35 | 0.7 | 56 / 56 | 0.9 | 75 / 75 | 1 | 87 / 88 | 0.5 |
| Co-prescriptions (%) |  |  |  |  |  |  |  |  |  |  |
| ACE-I/ARBs | 35 / 29 | 0.3 | 50 / 50 | 0.9 | 69 / 54 | 0.3 | 74 / 74 | 1 | 88 / 86 | 0.2 |
| Aspirin | 32 / 32 | 0.9 | 66 / 70 | 0.3 | 90 / 86 | 0.1 | 96 /95 | 0.6 | 99 / 99 | 0.5 |
| Beta-blockers | 17 / 16 | 1 | 36 / 37 | 0.8 | 59 / 59 | 0.9 | 78 / 78 | 0.9 | 93 / 91 | 0.4 |
| Statin-Adjuncts | 4 / 2 | 0.4 | 8 / 6 | 0.5 | 10 / 9 | 0.6 | 20 /16 | 0.09 | 44 / 37 | **0.02** |

^a^Treated refers to patients on high-intensity statin therapy. Control refers to patients on moderate- or low-intensity statin therapy. CAD, coronary artery disease; CHF, congestive heart failure; CKD, chronic kidney disease; PAD, peripheral artery disease; MACE, major adverse cardiac event; ACE-I, angiotensin-converting enzyme inhibitor; ARBs, angiotensin II receptor blockers.
