# Supplementary material for: Statin Intensity or Achieved LDL? Practice-based Evidence for the Evaluation of New Cholesterol Treatment Guidelines
Source: PLoS One. 2016 May 26;11(5):e0154952. doi: 10.1371/journal.pone.0154952 (PMC4881915; doi:10.1371/journal.pone.0154952)
Supplement: S5 Table — aTreated refers to patients on high-intensity statin therapy. Control refers to patients on moderate- or low-intensity statin therapy. LDL, low-density lipoprotein; HDL, high-density lipoprotein. (DOCX) [file pone.0154952.s006.docx]

S5 Table. Cholesterol profiles of patients treated with high intensity statins versus low or moderate intensity

statins by strata (N = 7,373)

|  | Strata 1  N= 1,475  Treated^a^/Control | Strata 2  N= 1,474  Treated/Control | Strata 3  N= 1,475  Treated/Control | Strata 4  N= 1,474  Treated/Control | Strata 5  N= 1,475  Treated/Control |
| --- | --- | --- | --- | --- | --- |
| Achieved LDL (mg /dL) | 105 / 111 | 94 / 101 | 89 / 94 | 85 / 89 | 77 / 83 |
| Achieved HDL (mg/dL) | 57 / 58 | 51 / 53 | 50 / 51 | 49 / 49 | 46 / 45 |
| Achieved Triglycerides (mg/dL) | 127 / 123 | 139 / 128 | 128 / 125 | 123 / 127 | 125 / 128 |

**^a^**Treated refers to patients on high-intensity statin therapy. Control refers to patients on moderate- or low-intensity statin therapy; LDL, low-density lipoprotein; HDL, high-density lipoprotein.
