# Supplementary material for: Statin Intensity or Achieved LDL? Practice-based Evidence for the Evaluation of New Cholesterol Treatment Guidelines
Source: PLoS One. 2016 May 26;11(5):e0154952. doi: 10.1371/journal.pone.0154952 (PMC4881915; doi:10.1371/journal.pone.0154952)
Supplement: S6 Table — aAdjusted for age, gender, race, ethnicity, history of coronary artery disease, congestive heart failure, chronic kidney disease, hypertension, peripheral artery disease, Type 2 diabetes, and co-prescriptions including angiotensin-converting enzyme inhibitors/angiotensin II receptor blockers, aspirin, beta-blockers, and statin adjuncts. bScaled variable. Center LDL = 95 ± 31. Therefore for each 31 mg/dL increase in achieved LDL the hazard rate for MACE increases by 10%. cScaled variable. Center HDL = 51 ± 16. dScaled variable. Center Triglyceride = 127 ± 79 mg/dL. MACE, major adverse cardiac event; LDL, low-density lipoprotein; HDL, high-density lipoprotein. (DOCX) [file pone.0154952.s007.docx]

S6 Table. Stratified Cox proportional hazards model^a^ of MACE Outcomes based on patient characteristics

|  | Hazard Ratio [95% CI] | P-value |
| --- | --- | --- |
| Cholesterol |  |  |
| Achieved LDL^b^ | **1.1 [1.05, 1.2]** | **0.0004** |
| High-Intensity Treatment | 0.9 [0.7, 1.1] | 0.2 |
| Moderate-Intensity Treatment  Low-Intensity Treatment | 1.0 [0.9, 1.2]  [Reference] | 0.5 |
| HDL^c^ | 1 [0.9, 1] | 0.6 |
| Triglycerides^d^ | 1 [0.96, 1.1] | 0.4 |
| Clinical Factors |  |  |
| Congestive Heart Failure | **1.2 [1.01, 1.4]** | **0.04** |
| Aspirin Therapy | **0.8 [0.7, 0.99]** | **0.047** |

^a^Adjusted for age, gender, race, ethnicity, history of coronary artery disease, congestive heart failure, chronic kidney disease, hypertension, peripheral artery disease, Type 2 diabetes, and co-prescriptions including angiotensin-converting enzyme Inhibitors/angiotensin II receptor blockers, aspirin, beta-blockers, and statin adjuncts. ^b^Scaled variable. Center LDL = 95 ± 31. Therefore for each 31 mg/dL increase in achieved LDL the hazard rate for MACE increases by 10%. ^c^Scaled variable. Center HDL = 51 ± 16. ^d^Scaled variable. Center Triglyceride = 127 ± 79 mg/dL. MACE, major adverse cardiac event; LDL, low-density lipoprotein; HDL, high-density lipoprotein.
